# PRKAR1A is a functional tumor suppressor inhibiting ERK/Snail/E-cadherin pathway in lung adenocarcinoma

**DOI:** 10.1038/srep39630

**Published:** 2016-12-20

**Authors:** Shaoqiang Wang, Yuanda Cheng, Yingying Zheng, Zhiwei He, Wei Chen, Wolong Zhou, Chaojun Duan, Chunfang Zhang

**Affiliations:** 1 Department of Thoracic Surgery, Xiangya Hospital, Central South University, Xiangya Road 87th, Changsha, 410008, Hunan, P. R. China; 2 Department of endocrinology, Xiangya Hospital, Central South University, Xiangya Road 87th, Changsha, 410008, Hunan, P. R. China; 3 Institute of Medical Sciences, Key Laboratory of Cancer Proteomics of Chinese Ministry of Health, Xiangya Hospital, Central South University, Xiangya Road 87th, Changsha, 410008, Hunan, P. R. China

## Abstract

Protein Kinase cAMP-Dependent Regulatory Type I Alpha (PRKAR1A) is a tissue-specific extinguisher that transduces a signal through phosphorylation of different target proteins. Loss of PRKAR1A was frequently observed in endocrine neoplasia and stromal cell tumors. However, a few cases were seen in epithelial tumors. Previously, we first found that PRKAR1A was downregulated in lung adenocarcinoma patients. Thus, the present study aimed to clarify its clinical implication and biological function as a tumor suppressor in lung adenocarcinoma. The low levels of PRKAR1A transcript were correlated with tumor progression and poor overall survival. The re-expression of PRKAR1A in H1299 cells suppressed the tumor cell proliferation and migration; stable knockdown (KD) of PRKAR1A in A549 cells enhanced this function both *in vitro* and *in vivo*. Moreover, KD of PRKAR1A in A549 cells promoted the statistical colonization of circulating tumor cells to the lungs in nude mice. These effects by PRKAR1A were attributed to inhibiting E-cadherin expression. Elevated E-cadherin significantly suppressed the PRKAR1A-KD induced cell proliferation and migration. Most notably, deletion of PRKAR1A inhibited E-cadherin by activating ERK/Snail signaling. In conclusion, PRKAR1A was a potent suppressor, and through the inhibition of PRKAR1A-ERK-Snail-E-cadherin axis could serve as a potential therapeutic target.

Lung cancer poses a major public health issue and is the most common cause of cancer-related deaths worldwide, especially, increasing throughout the developing countries^1^. It has been estimated that 1.8 million cases of tracheal, bronchus, and lung cancer were diagnosed in 2013, and 1.6 million deaths occurred^1^. The 95% of primary lung malignancies can be categorized into four major histological types including adenocarcinoma, squamous cell carcinoma, large cell carcinoma, and non-small cell lung cancer^2^. Adenocarcinoma, the most prevalent histological type, accounts for almost half of the primary lung cancer^3^. Despite advances in adenocarcinoma therapy (for example, surgery, chemotherapy, and radiotherapy), the average 5-year survival rate is dismal (approximately 18%)^4^. Moreover, up to 40% of the early stage patients develop local or distal metastasis^5^, which is the most devastating progression whose underlying mechanisms remain elusive. A majority of adenocarcinomas originated in the pulmonary epithelial cells and correlated with the inactivation of tumor-suppress genes or activation of the oncogene^6^.

PKA is a heterotetramer inactive kinase composed of two regulatory and two catalytic submits. The regulatory subunits are encoded by four genes (*PRKAR1A, PRKAR2A, PRKAR1B*, and *PRKAR2B). PRKAR1A*, a gene coding for the cAMP–dependent protein kinase (PKA) 1α regulatory submit, is located on human chromosome 17q22-24^7^. PRKAR1A protein insufficiency and PKA dysregulation have been implicated in various types of disorders, including Albright hereditary osteodystrophy (AHO), pseudohypoparathyroidism (PHP), acrodysostosis (ACRDYS)^8^, and Carney complex^9^. Interestingly, recent studies also showed that PRKAR1A protein expression level was significantly dysregulated in multiple primary carcinomas and distant metastases, such as cardiac myxomas^10^, odontogenic myxomas^11^, anaplastic thyroid carcinomas^12^, breast cancer^13^, pediatric pituitary adenomas^14^, and Schwann cell tumors^15^.

Previous studies strongly suggested that PRKAR1A dysregulation caused endocrine neoplasia, which was speculated to rely on tissues; however, its role in epithelium-generated tumors was rarely reported, especially in lung adenocarcinoma. Analyses of public databases revealed that PRKAR1A was downregulated in human lung adenocarcinoma. In order to elucidate the role of PRKAR1A in lung adenocarcinoma, we compared the expression of PRKAR1A mRNA and protein with the adjacent normal lung tissues. The clinical relevance of PRKAR1A has been significantly correlated with a tumor, lymph node, and metastasis stages (TNM). Furthermore, we investigated the correlation between the expression PRKAR1A and E-cadherin in order to explore the role of PRKAR1A in epithelial-mesenchymal transition (EMT) of lung adenocarcinoma proliferation and metastasis.

## Results

### Low level of tumor PRKAR1A expression was correlated with poor patient survival in lung adenocarcinoma

**Figure 1:**
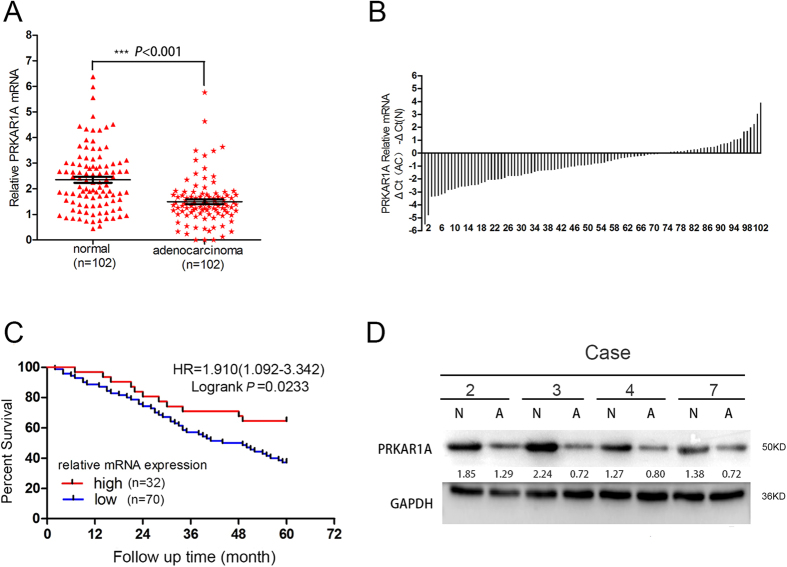
Downregulated PRKAR1A levels in lung adenocarcinoma samples and correlation with poor survival. (**A**) PRKAR1A was differently expressed in adenocarcinoma and normal tissue (*P* < 0.001). (**B**) The comparison of PRKAR1A expression from 102 adenocarcinoma patients’ cancerous lung tissues with the respective adjacent normal tissues. (**C**) Kaplan-Meier survival curves for the two groups of lung adenocarcinoma patients. The overall survival times in the low (blue, n = 70) and high PRKAR1A (red, n = 32) groups, with a hazard ratio of 1.91 (95% confidence interval (CI) 1.09–3.34) and *P* = 0.0233. Statistical significance was assessed by paired t-test (**A**) and Mantel-Cox log-rank test (**C**). (**D**) The decreased level of PRKAR1A protein was detected in 61 out of 102 tumor samples compared with the paired normal tissues (N, normal tissue; A, adenocarcinoma). The representative Western blot images of PRKAR1A was illustrated and normalized against GAPDH.

To determine the clinical significance of PRKAR1A in lung adenocarcinomas, we investigated the expression of PRKAR1A in 102 specimen pairs of frozen adenocarcinomas and cognate normal lung tissues which were 5 cm distant from the tumor margins. The quantitative reverse transcriptase PCR (qRT-PCR) revealed that the RNA level in adenocarcinomas was significantly decreased as compared to the adjacent normal samples (mean ± SE: 1.50 ± 0.09 vs. 2.35 ± 0.12, *P* < 0.0001) (Fig. 1A). Figure 1B further displayed that low PRKAR1A expression was observed in 68.63% cases (70/102). Interestingly, there were neither increased or decreased PRKAR1A mRNA expression in about 5% cases (5/102) compared to normal tissues. A threshold of 5-year follow-up demonstrated that patients with equal or low PRKAR1A expression (70 cases) had a poor overall survival compared to those with high expression (32 cases) (HR = 1.910, 95%CI 1.092–3.342, log-rank *P* = 0.0233) according to the Kaplan-Meier survival curve (Fig. 1C). There was an even greater overall decreased 5-year survival for patients with the lowest PRKAR1A (bottom quartile, n = 25) levels compared with that of patients with the highest (top quartile, n = 25) expression (HR = 2.590, 95% CI 1.212–5.533, log-rank *P* = 0.0136) (Figure S1B).

Furthermore, we compared the protein levels of PRKAR1A in clinically collected human lung adenocarcinoma specimens and paired adjacent normal tissues using immunoblotting (western blotting). Among 102 pairs of samples examined by WB, 61/102 samples (59.80%) showed decreased PRKAR1A protein compared with their corresponding normal tissues (Fig. 1D). Importantly, low PRKAR1A protein expression was also found to be associated with short overall survival in patients (HR = 1.880, 95% CI 1.136–3.113, log-rank *P* = 0.0141) (Figure S1C). Taken together, our data indicated that downregulation PRKAR1A expression profile correlates with poor prognosis in lung adenocarcinoma after the operation.

### PRKAR1A inhibited the proliferation and migration of lung adenocarcinoma cells

**Figure 2:**
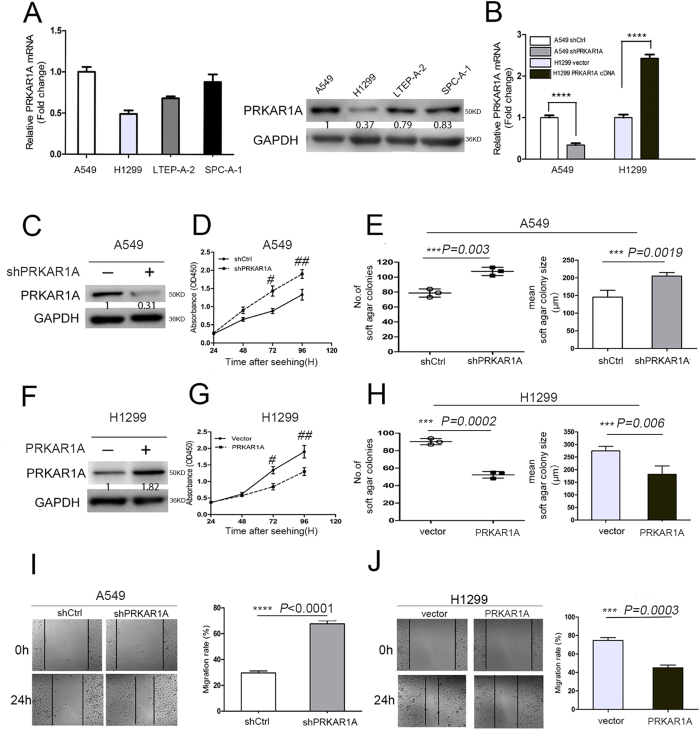
PRKAR1A inhibited the proliferation and migration of lung adenocarcinoma cells. (**A**) qRT-PCR and western blotting (WB) analysis (performed in triplicate) of PRKAR1A in human lung adenocarcinoma cells. (**B–J**) A549 cells or H1299 cells were stably transfected with shPRKAR1A or PRKAR1A, respectively, and screened by qRT-PCR and WB. (**B**) ****P < 0.001. (**D**,**G**) CCK-8 (Cell Counting Kit-8) and (**E,H**) soft agar assays (number and mean size of clonogenic colonies estimated after two weeks) were performed to analyze A549 or H1299 cells proliferation. (**D**) ^#^*P* = 0.0037, ^##^*P* = 0.0065; (**G**) ^#^*P* = 0.0048, ^##^*P* = 0.0039. (**I** and **J**) Scratch-wound assays were performed to analyze the migration of A549 and H1299 cells. Data were representative of three independent experiments.

We examined PRKAR1A expression by qRT-PCR and WB in four lung adenocarcinomic alveolar epithelial cell lines such as H1299, LTEP-A-2, SPC-A-1, and A549, which range from low- to high-level PRKAR1A expression. We found that PRKAR1A expression was relatively high in A549 cell and low in an H1299 cell line derived from metastatic lymph node (Fig. 2A). To elucidate the biological function *in vitro*, we stably generated A549 and SPC-A-1 cells expressing control shRNA (shCtrl) or distinct PRKAR1A shRNA (shPRKAR1A), which was confirmed by qPCR and WB (Fig. 2B,C, and Figure S2A). PKA activity was measured in the protein lysates from A549-shPRKAR1 and A549-shCtrl cells (Figure S3A). Analysis of the basal (unstimulated) and total (cAMP-induced) PKA activity revealed significantly higher baseline PKA activity in A549-shPRKAR1A cells as compared to the control cells (*P* = 0.0019). Accordingly, an elevated level of phosphorylated CREB, a canonical downstream target of PKA, was exhibited in A549-shPRKAR1A cells (Figure S3C).

CCK-8 assays to analyze A549 and SPC-A-1 cells’ proliferation (Fig. 2D and Figure S2B) displayed that PRKAR1A depletion strongly enhanced the cell viability. Similarly, the downregulation of PRKAR1A in A549 cells significantly increased the colony formation ability both in numbers and mean size of soft agar colonies (Fig. 2E, Figure S2D). Furthermore, we examined the effect of PRKAR1A downregulation on the migration capacity by scratch-wound assay, which demonstrated that a large number of A549-shPRKAR1A or SPC-A-1-shPRKAR1A cells filled in the wound space than the control cells (A549-shCtrl or SPC-A-1-shCtrl) 24h post wounding (Fig. 2I and Figure S2C).

Next, we selected H1299 cells, in which endogenous PRKAR1A was weakly expressed to transduce with either vector virus (pHY-LV-OE1.6) or pHY-LV-OE-PRKAR1A virus (henceforth abbreviated as PRKAR1A) (Fig. 2B,F). As expected, H1299-PRKAR1A cells showed decreased baseline PKA activity (*P* = 0.0025) and phosphorylated CREB as compared to H1299-vector cells (Figure S3B, C). Moreover, PRKAR1A overexpression suppress the viability of H1299 cells (Fig. 2G) and reduced the ability of colony formation (Fig. 2H, Figure S2E). In addition, the ectopic expression of PRKAR1A partially inhibited the migration ability of H1299 cells (Fig. 2J). Overall, our data suggested that PRKAR1A could inhibit the proliferation and migration of lung adenocarcinoma cells.

### Aberrant PRKAR1A expression influenced adenocarcinoma cells’ tumorigenesis in lung tissue

**Figure 3:**
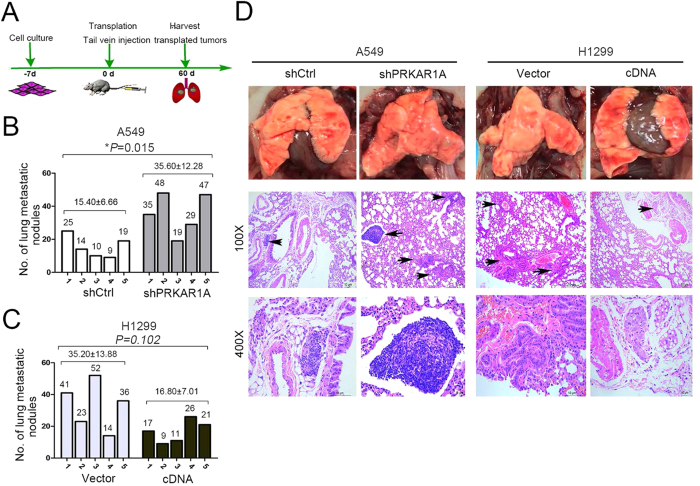
Aberrant PRKAR1A expression influenced adenocarcinoma cells’ tumorigenesis in the lung. (**A**–**D**) A549 or H1299 cells were infected with silenced (shPRKAR1A) or encoded PRKAR1A virus and compared with an empty retrovirus (shCtrl or vector). Cells were injected into the tail vein of nude mice. (**A**) Schematic of the *in vivo* tumorigenesis assay in the lung. (**B** and **C**) Number of metastasis of each nude mice. (**D**) Representative images of the gross lung [**D**, upper], and H&E stained lung sections, with arrows pointing to tumors. n = 5 for each group, data were mean ± SD.

In order to elucidate the function of PRKAR1A in tumorigenesis *in vivo*, an experimental metastasis assay was utilized to compare the metastatic tumor nodules formed in the lungs of nude mice. These mice in two different groups were administered with A549-shPRKAR1A or H1299-PRKAR1A cells (controls received A549-shCtrl or H1299-vector individually) by tail vein inoculation. After 60 days, pulmonary surface nodules and microscopic lesions in the lungs were observed (Fig. 3D), and the mice were executed by cervical dislocation, subjected to necroscopy, and lungs harvested (Fig. 3A). After 60 days of A549-shPRKAR1A or H1299-vector induction, the lung lesions were larger compared with those from control A549-shCtrl or H1299-PRKAR1A, respectively (Fig. 3D). The gross number of metastatic nodules in the lungs was significantly more in mice injected with A549-shPRKAR1A cells than with A549-shCtrl cells (35.60 ± 12.28 vs. 15.40 ± 6.66; *P* = 0.015; Fig. 3B,D). As anticipated, in H1299 matched-pair mice, the total number of lung lesions was fewer in H1299-PRKAR1A mice than the control but did not achieve statistical significance (16.80 ± 7.01 vs. 35.20 ± 13.85; *P* = 0.102; Fig. 3C,D).

### The expression between PRKAR1A and E-cadherin were positively correlated in lung adenocarcinoma patients

**Figure 4:**
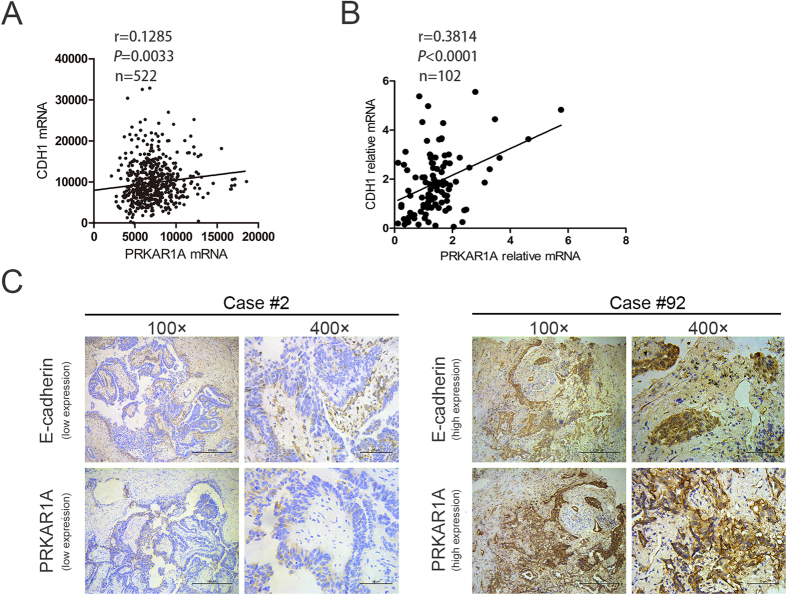
The expression of PRKAR1A and E-cadherin was positively correlated in lung adenocarcinoma patients. (**A**) Correlation analysis of PRKAR1A level and CDH1 mRNA in TCGA dataset of 522 lung adenocarcinoma patients (r = 0.1285, *P* = 0.0033). (**B**) Correlation analysis of PRKAR1A level and CDH1 mRNA in 102 lung adenocarcinoma patients from XiangYa Hospital lung tumor tissue bank who underwent surgical resection (r = 0.3814, *P* < 0.0001). (**C**) Representative images of immunohistochemical staining for PRKAR1A and E-cadherin in human adenocarcinoma tissues.

Analysis of a clinical patient database, the cancer genome atlas (TCGA), by cBioPortal (http://www.cbioportal.org/public-portal/), demonstrated a weak positive correlation between PRKAR1A mRNA expression and CDH1 mRNA (encoding E-cadherin protein) level in 522 lung adenocarcinoma patients (r = 0.1285; *P* = 0.0033; Fig. 4A). Next, we examined the correlation between PRKAR1A and E-cadherin mRNA and protein level by analyzing 102 lung adenocarcinoma tissues from our lung tumor tissue repository, of all the patients who underwent surgical resection. PRKAR1A mRNA was correlated with CDH1 expression in lung adenocarcinoma tissues (r = 0.3814, *P* < 0.0001; Fig. 4B). Then, we assessed the expression levels of PRKAR1A and E-cadherin proteins in the same 102 lung adenocarcinoma specimens by immunohistochemistry (Fig. 4C, Figure S4). The correlation between the clinicopathological characteristics of 102 lung adenocarcinoma patients and the expressions of PRKAR1A and E-cadherin were summarized in Table 1. PRKAR1A and E-cadherin been inversely correlated with patients’ TNM stage (*P* < 0.05). Table 2 showed that the expression of PRKAR1A was positively correlated with E-cadherin in 102 lung adenocarcinoma tissues (*P* = 0.029).

### PRKAR1A low-expression strongly aggravated the decreased expression of E-cadherin and the mesenchymal phenotype, rescued by E-cadherin overexpression

**Figure 5:**
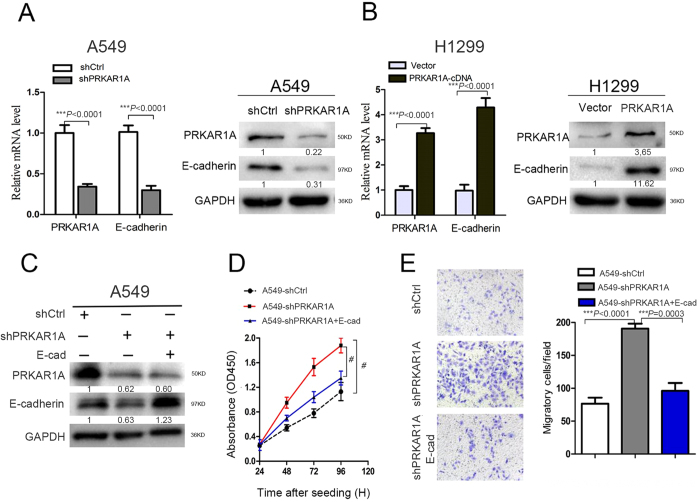
E-cadherin reversed the effect of PRKAR1A downregulation on cell growth and migration *in vitro*. (**A** and **B**) PRKAR1A regulated the E-cadherin expression in A549 and H1299 cells as confirmed by qRT-PCR and WB, mean ± SD, n = 3. (**C–E**) A549 cells were stably transfected with shPRKAR1A and transiently transfected with CDH1-plasmid into a portion of parental A549-shPRKARA1 cells. (**C**) The protein levels of PRKAR1A and E-cadherin were determined by WB. (**D**) Cell proliferation was determined by CCK8 assay, n = 3, bar: SD. (**E**) The transwell migration assay was conducted to quantify the migrated cells. n = 3, bar: SD. The band in western blotting was normalized to GAPDH and the corresponding cells.

E-cadherin has been shown to be weakly expressed during progression in a broad range of tumors originating in epithelial cells^16^. Decreased mRNA and protein levels of PRKAR1A were observed in adenocarcinoma tissues, thereby instigating an *in vitro* experiment to assess if PRKAR1A was required for E-cadherin expression. Herein, we knocked down the PRKAR1A expression in A549 cells and upregulated PRKAR1A in H1299 cells. Knockdown of PRKAR1A resulted in an approximately four-fold diminution of E-cadherin expression at both mRNA and protein levels in A549 cells (Fig. 5A). Furthermore, this positive regulatory function was confirmed in H1299 cells (Fig. 5B). Taken together, this study provided strong evidence that PRKAR1A positively regulated E-cadherin expression in human lung adenocarcinoma.

E-cadherin is a candidate tumor suppressor gene. Downregulation of E-cadherin is a hallmark of EMT, which has been established as a critical event for early metastatic stages, leading to loss of cell-cell interactions and acquisition of invasive behavior^16^,^17^,^18^. Therefore, to explore whether elevated expression of E-cadherin could contribute to reverse the A549-shPRKAR1A cells proliferation and metastases, we transiently transfected pcDNA3/E-cadherin plasmid (abbreviated as E-cad) into a portion of parental A549-shPRKARA1 cells (Fig. 5C). Interestingly, we observed slightly different PRKAR1A expression between the reconstituted E-cadherin overexpressing A549-shPRKAR1A cells and the parental A549-shPRKAR1A cells (Fig. 5C). However, the overexpression of E-cadherin in A549 cells dramatically blocked the proliferation and migration induced by the loss of PRKAR1A expression (Fig. 5D,E). These data indicated that E-cadherin was a downstream effector in the process of PRKAR1A-induced inhibition of lung adenocarcinoma cell proliferation and migration.

### PRKAR1A regulated E-cadherin expression through ERK/Snail/E-cadherin signaling

**Figure 6:**
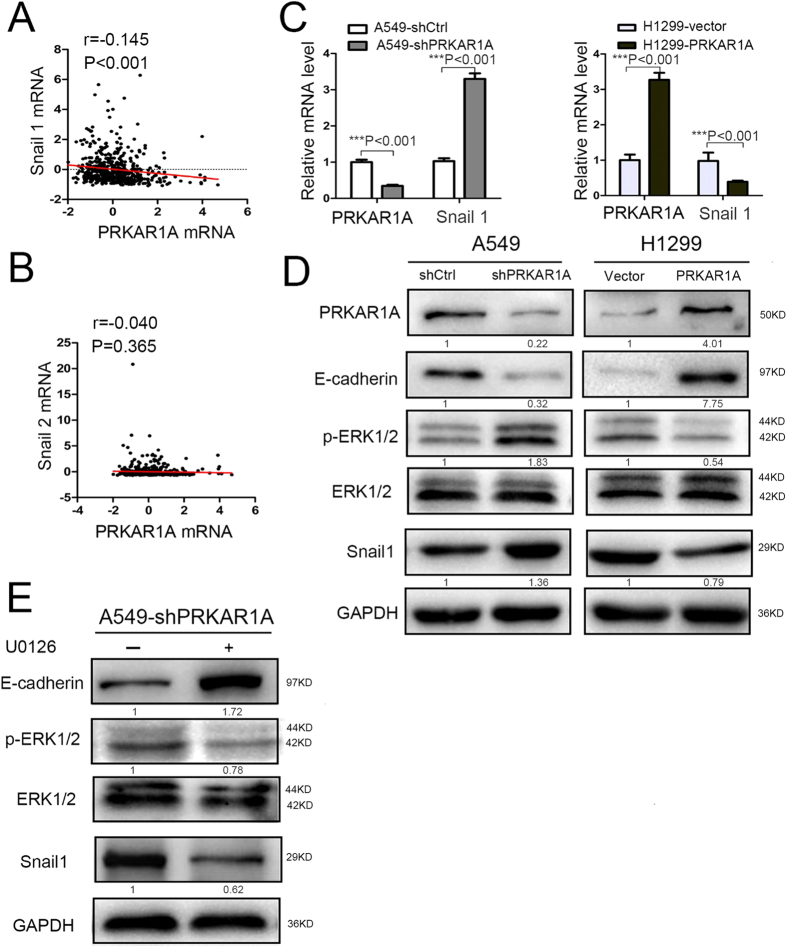
PRKAR1A regulated E-cadherin expression through ERK/Snail/E-cadherin signaling. (**A–C**) PRKAR1A inhibited the expression of Snail1. (**A** and **B**) The correlation between PRKAR1A and Snail1 or Snail2 was analyzed by Pearson’s correlation. The mRNA data was retrieved from TCGA by cBioPortal, n = 522. (**C** and **D**) qRT-PCR showed that PRKAR1A affected the expression of Snail1. n = 3, bar: SD. (**D**) Phospho-ERK1/2 detected in A549 and H1299 cells either downregulated or upregulated the *PRKAR1A* gene. The bands of p-ERK1/2 were normalized to ERK protein. (**E**) A549 cells with stable PRKAR1A knockdown were treated by ERK inhibitor U0126 for 24 h. WB was assessed detect the expression of E-cadherin, p-ERK1/2, and Snai1 (encoded by *Snail*). p–ERK1/2 band normalized by ERK, E-cadherin, and Snai1 bands normalized to GAPDH.

Loss of E-cadherin/CDH1 function has been hypothesized to contribute to cancer progression by increasing proliferation, invasion, and/or metastasis^19^,^20^. Enhanced expression of Snail superfamily, especially Snail1 and Snail2, has been an emerging hallmark of a subset of cancers. It is characterized by binding to E-box of E-cadherin/CDH1 promoter to repress E-cadherin transcription and induce EMT^21^. To test if PRKAR1A also regulated E-cadherin by inhibiting Snail (Snail1 and/or Snail2) expression, cBioPortal for TCGA was utilized for reanalysis. The Pearson correlation analyses of the microarray data showed that PRKAR1A mRNA levels had inversely weak correlation with the mRNA levels of Snail1 (encoding snail1), but not Snail2 (encoding slug) (Fig. 6A,B). Next, we assessed the effect of PRKAR1A knockdown or upregulation on Snail1 levels in A549 or H1299 cells, respectively. The qRT-PCR and WB results showed that PRKAR1A knockdown significantly increased the average Snail1 expression (Fig. 6C,D), whereas the inverse correlation between PRKAR1A and Snail1 was detected in transfected H1299 cells (Fig. 6C,D). Notably, these phenotypes have been concomitant with the above-described alteration in E-cadherin expression.

Snail was reported as a critical downstream effector of MAPK/ERK pathway at a transcript level^21^,^22^. To detect the adequate amount level of PRKAR1A expression required for the upregulation of the extracellular signal kinase (ERK)1/2, which is frequently involved in the regulation of lung adenocarcinoma cell proliferation and migration^23^,^24^, we tested the phosphorylation level of ERK1/2 in PRKAR1A knockdown or upregulated cells by WB. As compared with the control cells, phospho-ERK1/2 was moderately increased upon PRKAR1A-knockdown in A549 cells and decreased in PRKAR1A-upregulated H1299 cells (Fig. 6D).

To further elucidate the function of ERK1/2 in PRKAR1A-induced EMT phenotype, A549-shPRKAR1A cells were treated by H89, an inhibitor of PKA. p-ERK1/2 levels decreased in H89-treated cells as compared to the untreated H89 cells (Figure S5). Subsequently, the PRKAR1A-knockdown A549 cells were used treated by U0126, a well-characterized inhibitor of ERK1/2 (Fig. 6E). The phospho-ERK1/2 was downregulated upon PRKAR1A knockdown in U0126-stimulated cells and H89-stimulated cells, thereby significantly inhibiting the expression of Snail1 and upregulated E-cadherin (Fig. 6E). Collectively, our data suggested that insufficient PRKAR1A induced cell proliferation and metastasis by E-cadherin downregulation, which was mediated through activated ERK/Snail pathway.

## Discussion

Aberrant PRKAR1A expression has been linked to increased tumorigenesis and metastasis in multiple endocrine neoplasia^10^,^12^,^25^,^26^. Additionally, in mesenchymal cells, PRKAR1A deletion also led to tumorigenesis in bone tissues^27^,^28^. However, its role in epithelial cancers was scarcely reported. The conflicting data for low PRKAR1A/high SRC expression was associated with poor clinical outcome in human breast cancer derived from epithelium^13^; however, PRKAR1A was overexpressed in cholangiocarcinoma (CCA)^29^. The previous studies indicated that PRKAR1A could determine the tissue specificity and/or context-dependence of some miRNAs^30^,^31^. Our data demonstrated that PRKAR1A fits the putative definition of an anti-oncogene in the lung and that it regulated ERK/Snail signaling pathway in lung adenocarcinoma cells. This result offered critical insight into the contribution of the molecule towards lung tumorigenesis, thereby markedly widening the knowledge of PRKAR1A in lung adenocarcinoma. Compared with normal lung tissues, the primary lung adenocarcinomas exhibited low PRKAR1A levels. The expression of PRKAR1A decreased with tumor progression, the size of tumors, lymph node metastasis, and also with the higher stages (III, IV) of adenocarcinomas. Contrary to A549 cells, H1299 cells derived from metastatic lymph node expressed less. This provided an additional clarification for the correlation of PRKAR1A expression with tumor progression and metastasis.

We tested the classic tumor proliferation and metastasis function of PRKAR1A in lung adenocarcinoma cells by both *in vitro* and *in vivo* assays. Our data showed that PRKAR1A deletion increased the proliferation and migration of lung adenocarcinoma cells and that decreased levels of PRKAR1A led E-cadherin upregulation to reasonably conjecture that PRKAR1A was an anti-oncogene in the lung. Moreover, we examined whether PRKAR1A alteration could contribute to tumor formation and growth *in vivo*, especially in the lung. We used shRNA, for the first time, to inactivate PRKAR1A in lung adenocarcinoma cells and observed that PRKAR1A deletion enhanced *in vivo* lung metastasis in nude mice. Importantly, in our clinicopathological analysis, a positive correlation was established between PRKAR1A and E-cadherin in lung adenocarcinomas. Notably, the loss of E-cadherin was a critical marker for EMT^32^. E-cadherin has been known to suppress tumor cell migration in a series of malignancies^33^,^34^. And E-cadherin can affect the cell growth in a number of ways, including cell-cell contact inhibition^35^,^36^,^37^ and independent cell-cell interactions modulating growth inhibitory signals^38^. We demonstrated that elevated E-cadherin in shPRKAR1A-induced cells could partially relieve proliferation and migration, thereby extrapolating that E-cadherin was an essential target for PRKAR1A in the regulation of lung adenocarcinoma cells’ proliferation and metastatic behavior.

E-cadherin inactivation was frequently mediated by transcriptional or translational mechanisms^20^,^39^. The loss of E-cadherin is a key event in EMT. Several important EMT drivers such as Snail1 and Snail2 have been shown to correlate with cancer relapse and survival. The Snail factors bind E-box consensus sequences in E-cadherin promoter to suppress its expression^20^,^32^,^40^. The TCGA microarray data revealed that Snail1 in PRKAR1A low-expression lung adenocarcinoma patients did not show elevated Snail2. *In vitro* PRKAR1A-knockdown and H89-untreated A549 cells, elevated Snail1 expression both at mRNA and protein levels was observed with a concomitant decrease in E-cadherin expression. Therefore, we speculated that PRKAR1A induces EMT by regulating Snail1. In our vitro experiments, we further elucidated that PRKAR1A deficiency up-regulated snail expression by the activation of ERK pathway, which was critical for the initiation and progression of numerous cancers^41^,^42^,^43^. ERK kinase could activate Snail to further regulate EMT^44^,^45^. Collectively, through the modulation of ERK/Snail/E-cadherin signaling pathway, *PRKAR1A* could act as a tumor suppressor gene by inhibiting lung adenocarcinoma growth and metastasis. This phenomenon was similar to other studies in breast cancers^13^ and contrary to the function in CCA^29^. Therefore, our findings further suggested a multi-faceted role of PRKAR1A in tumorigenesis dependent on tissue specificity.

In summary, the present study revealed the biological function and clinical significance of PRKAR1A as a suppressor in lung adenocarcinoma. Our results provided an impetus to further investigate PRKAR1A in ERK/Snail signaling in lung cancer. The pharmaceutical intervention of PRKAR1A might provide a promising treatment to alleviate lung adenocarcinoma progression.

## Methods

### Patient samples

Fresh tumor specimens and paired adjacent non-tumor tissues were obtained by radical resection from those lung adenocarcinoma patients without any antitumor therapeutic intervention at the thoracic department, Xiangya Hospital, Central South University (CSU). The tumor-node-metastasis was classified based on the criteria of the seventh tumor-node-metastasis (TNM) staging system. The cohort of 102 lung adenocarcinoma patients provided written informed consent. The collection and usage of the clinical specimens were approved by the Xiangya Hospital Medical Research Ethics Committee. The experimental methods were carried out according to those approved by the Scientific Research Project 201403216 (Histopathological Application) of Xiangya Hospital.

### Immunohistochemistry (IHC)

10% Formalin-fixed and paraffin-embedded tissue sections (4 μm) were stained with polyclonal rabbit anti-PRKAR1A (1:100, ab139695, Abcam, Cambridge, MA, USA) and anti-E-cadherin antibody (1:500, ab40772, Abcam, Cambridge, MA, USA). The expression scores assigned (staining intensity: 0, no staining; 1, light yellow; 2, yellow; 3, brownish yellow) were multiplied by the proportion of the stained cells (0, <5% stained; 1, 6–25% stained; 2: 26–50%; 3: ≥50%). We evaluated protein expression using staining index (SI), which was calculated of the proportion of positive cells and the staining intensity score as possible total scores of 0, 1, 2, 3, 4, 6 and 9. The negative expression of PRKAR1A was defined as <3, and ≥3 was defined as a positive expression. All IHC stained slides were scored by two independent doctors of pathology, and clinical features and average values were used for the final PRKAR1A and E-cadherin scores.

### Cell culture, lentiviral production, plasmid, and infection

Four lung adenocarcinoma cell liens (A549, H1299, SPC-A-1, LTEP-A-2) were purchased from the Chinese Academy of Sciences Cell Bank (Shanghai, China). All lung adenocarcinoma cell lines were cultured in RPMI 1640 supplemented with 10% FBS and 100 U/mL penicillin/streptomycin (GIBCO BRL Co., MD, USA). PRKAR1A was knocked down in A549 cells by pHY-LV-KD5.1 lentiviral construct (puromycin selection marker) (Hanyin Co, Shanghai, China). PRKAR1A cDNA (NM_212472.1) was purchased form Biovector Science Lab, Inc (Beijing, China), and primers designed at the beginning and end of PRKAR1A CDS coding regions were synthesized by Sangon Biotech, Inc (Shanghai, China). The sequences of the PRKAR1A primers were: PRKAR1A-P1 (EcoRI) CCGCTCGAGGCCACCATGGAGTCTGGCAGTACCGCCG; PRKAR1A-P2 (BamHI) CGGGATCCTCAGACAGACAGTGACACAAAACTGTTG. Then, corresponding Restriction Enzyme (EcoRI and BamHI) cutting sites are added on the 5′end of primer. Amplify the target gene fragment by taking cDNA as a template. After the target fragment is recovered by running gel, Restriction Enzyme cutting sites on two ends are used to process the fragment and purify the recovery. Use the same Restriction Enzyme cutting sites to process pHY-LV-OE1.6 vector (puromycin selection marker) (Hanyin Co, Shanghai, China), so that it is linearized. After the vector is recovered by the gel, connect with the fragment and transform the DH5α competent cell. Identify the positive clone through PCR and test the sequences of PRKAR1A.

Subsequently, lentiviral was used for assembly and transduction into H1299 cells stably overexpressing PRKAR1A. SYBR Green qRT-PCR and western blotting (see below) were used to verify PRKAR1A expression in all the cells. pcDNA3/E-cadherin plasmid containing human E-cadherin cDNA (NM_004360.3) was purchased from Addgene (#45769, Cambridge, MA, USA). pcDNA3/E-cadherin plasmid was transiently transfected into a portion of parental A549-shPRKARA1 cells according to the Lipofectamine^TM^ 2000 (Invitrogen, Carlsbad, CA, USA) protocol. After 18 hours, part of the transiently transfected cells were collected and screened for E-cadherin protein expression by western analysis. ERK inhibitor (U012) and PKA inhibitor (H89) were purchased from Selleckchem (Shanghai, China).

### Gene knockdown and overexpression

Lentiviral vectors for human PRKAR1A knockdown or PRKAR1A expression, carrying a green fluorescent protein (GFP) sequence, were constructed by Hanyin. The recombinant PRKAR1A-shRNA lentivirus (target sequence: 5′-GGGAATACTTGAGAGGTT-3′) (or PRKAR1A-expression) and the negative control lentivirus (GFP-lentivirus, target sequence: 5′-GTAGCGCGGTGTATTATAC-3′) were prepared and titrated to 10^9^TU/mL (transfection unit).

To obtain a stable PRKAR1A-knockdown (or PRKAR1A-overexpression) cell line, A549 (or H1299) cells were seeded in 6-well dishes at a density of 2 × 10^5^cells/per well. Subsequently, the cells were infected with the same titer virus with 8 μg/mL polybrene on the following day. Approximately 72 h after viral infection, GFP expression was confirmed under a fluorescence microscope, and the culture medium was replaced with selection medium containing 4 μg/mL puromycin. The cells were then cultured for a minimum of fourteen days. The puromycin-resistant cell clones were isolated, amplified in medium containing 2 μg/mL puromycin for seven to nine days, and transferred to a medium without puromycin. The clones were designated as A549-shPRKAR1A (knock down) or A549-shCtrl (negative control) cells and H1299-PRKAR1A (overexpression) or H1299-vector (negative control) cells.

### RNA purification, quantitative real-time PCR (qRT-PCR)

RNA was isolated using TRIzol (Invitrogen, Carlsbad, CA, USA) according to the manufacturer’s protocol. The expression level was quantified using SYBR Green Assay Kit (# RR820A, Takara Bio Inv, Otsu, Shiga, Japan). The following pairs of primers were used: PRKAR1A (F): 5′-GTA GCT GAT GCA TTG GAA CCAG-3′, (R): 5′-CCA ATC TTC CCA CTT CAA ACT-3′; E-cadherin (F): 5′-TGC CCA GAA AAT GAA AAA GG-3′, (R): 5′-GTG TAT GTG GCA ATG CGT TC-3′; Snail1 (F): 5′-CCT CAA GAT GCA CAT CCG AAG-3′, (R): 5′-ACA TGG CCT TGT AGC CA-3′; GAPDH (F): 5′-CCA GCA AGA GCA CAA GAG GAA-3′, (R): 5′-ATG GTA CAT GAC AAG GTG CGG-3′ (Takara design and synthesis). GAPDH was utilized to normalize mRNA.

### Western blotting

Whole-cell lysates were prepared from human lung adenocarcinoma cell lines after harvesting and centrifuging. The proteins were denatured, electrophoresed (SDS-PAGE gel Kit, Beyotime, Shanghai, China), and transferred to PVDF membrane (Millipore Co., MA, USA). Subsequently, the membranes were blocked with non-fat milk powder, incubated with primary and secondary (HRP-labeled goat anti-rabbit IgG) antibodies, and visualized (Bio-Rad Image Lab Software). The primary antibodies against PRKAR1A (1:1000, ab139695, Abcam, Cambridge, MA, USA), E-cadherin (1:3000, ab40772, Abcam), p-ERK1/2 (1:1000, 9101, Cell Signaling Technology (CST), Danvers, MA, USA), ERK1/2 (1:1000, 9108, CST), Snail (1:1000, 3879, CST), phosphorylated CREB (pCreb, Ser133, 1:1000, CST), and GAPDH (1:3000, Sangon, Shanghai, China) were used. For all PRKAR1A target genes, the intensity of each band was normalized to GAPDH. For p-ERK1/2, quantification was relative to the total ERK1/2. The quantification of protein bands was performed using Image J software.

### PKA activity assay

The SignaTect cAMP-Dependent Protein Kinase (PKA) Assay system (Promega, Sunnyvale, CA, USA) was used to measure the baseline (cAMP-untreated) and total (cAMP-treated) activity of PKA from the cells’ protein lysates, as described previously^13^. 50 μg protein lysate containing ^32^P-labeled phosphate was incubated with biotinylated kemptide (a PKA-specific substrate), in the absence or presence of 0.01 mM cAMP. The protein kinase inhibitor (PKI, 20 μM) was used to assess the PKA specificity. Perforated SAM2 biotin capture membranes were utilized to bind and immobilize the ^32^P-labeled substrate. The PKA activity was measured by standard scintillation to determine radioactive counts/min. All estimations of the PKA activity were repeated thrice for each cell line.

### Cell proliferation assay

2000cells/well were seeded in 96-well plates. Cell Counting Kit (CCK8) assays were performed according to the manufacturer’s instructions (CK04, Dojindo subsidiary, Shanghai, China). 10 μL CCK8 was added to per 100 μL culture and incubated at 37 °C for 2 h. 48 h after plating the proliferation was estimated at 450 nm at 24 h, 48 h, and 72 h.

For colony formation assay, 500cells/well were mixed with medium containing 0.4% agarose (low gelling temperature) and seeded in 24-well plates at low density. After two weeks, colonies larger than 100 μm in diameter were counted using an inverted microscope (Olympus CKX41).

### Cell migration assay

Scratch-wound assays were performed in triplicates in 6-well plates. The cells were grown as monolayers without creating any scratch-wound until confluency. The cell migration was imaged and the width of the wound measured.

Transwell assays (Corning Costar, NY, USA) were performed in triplicates using uncoated plates to assess the transformed and control cells’ migration. The cells were seeded into the upper chamber of transwell (5 × 10^4^ cells/well). The lower chamber was filled with RPMI 1640 containing 10% FBS (Gibco, BRL, USA). The chamber assembly was incubated at 37 °C with 5% CO_2_ for 6 h. The migrating cells were fixed with 4% paraformaldehyde for 15 min, stained for 30 min with crystal violet, and photographed under an inverted microscope (Olympus CKX41).

### *In vivo* metastasis assays

Stable PRKAR1A-low-expressing control A549 cells (1 × 10^6^ cells in 0.1 mL PBS) were injected intravenously into the tail vein into nude mice (4-week-old). All the nude mice were sacrificed 60 days after injection according to the procedures by the Animal Ethics Committee of Xiangya Hospital. The lungs were excised and embedded in paraffin for HE (hematoxylin and eosin) staining. We calculated the metastatic nodules by visible pulmonary surface nodules and microscopic nodules. First counted metastatic nodules to the pulmonary surface manually. The lung divided into left lobe and right lobe, then were embeded in paraffin for HE staining. Each lobe (left or right) was cut into five discrete pieces. Quantify the microscopic metastasis by statistically 5 animals per group &5 slides per lobe & 2 (left and right lobe per lung). The total number of metastatic nodules of each mouse was calculated by visible pulmonary surface nodules and microscopic nodules. The protocols followed were approved in accordance with the Scientific Research Project 201403217 (animal experiments) of Xiangya Hospital.

### Analysis of TCGA lung adenocarcinoma data

mRNA expression in lung adenocarcinoma was obtained from the TCGA (The Cancer Genome Atlas) data (TCGA Provision, 522 samples) via the cBioPortal (http://www.cbioportal.org/public-portal/).

### Statistics

Values were represented as mean ± SD, and 2-tailed paired t*-*test was used for two preselected groups. The χ^2^-test was used for the comparison of patients’ clinical characteristics. Pearson’s correlation determined the association between PRKAR1A and target mRNA expression. The overall survival times after lung resection were evaluated by Kaplan-Meier survival curve and log-rank test. *P* < 0.05 was considered statistically significant.

## Additional Information

**How to cite this article**: Wang, S. *et al*. PRKAR1A is a functional tumor suppressor inhibiting ERK/Snail/E-cadherin pathway in lung adenocarcinoma. *Sci. Rep.* **6**, 39630; doi: 10.1038/srep39630 (2016).

**Publisher's note:** Springer Nature remains neutral with regard to jurisdictional claims in published maps and institutional affiliations.

## Supplementary Material

Supplementary Figures

## Figures and Tables

**Table 1:** The correlation between PRKAR1A and E-cadherin immunostaining and clinicopathological features in 102 cases of lung adenocarcinoma tissues (χ^2^-test).

Variables	Case No.	PRKAR1A			E-cadherin		
		<3	≥3	*P*	<3	≥3	*P*
Age (year)							
<60	53	33	20	0.291	26	27	0.238
≥60	49	36	13		30	19	
Gender							
male	61	37	24	0.066	32	29	0.545
female	41	32	9		24	17	
Smoking							
yes	64	46	18	0.277	36	28	0.837
no	38	23	15		20	18	
Differentiation							
Well and moderate	67	46	21	0.825	30	37	*0.006
Poor and undifferentiation	35	23	12		26	9	
TNM stage							
I–II	66	39	27	*0.015	30	36	*0.012
III–IV	36	30	6		26	10	
Size of tumor							
<3 cm	43	24	19	*0.034	12	34	*0.000
≥3 cm	59	45	14		47	12	
Node metastasis							
no	30	8	22	*0.000	7	23	*0.000
yes	72	61	11		49	23	

**Table 2:** The correlation between expression levels of PRKAR1A and E-cadherin in 102 cases of lung adenocarcinoma cancer tissues by immunohistochemistry (χ^2^-test).

PRKAR1A	Case No.	E-cadherin		
		<3	≥3	*P*-value
<3	69	43	26	**0.029**
≥3	33	13	20	
