# Supplementary material for: PRKAR1A is a functional tumor suppressor inhibiting ERK/Snail/E-cadherin pathway in lung adenocarcinoma
Source: Sci Rep. 2016 Dec 20;6:39630. doi: 10.1038/srep39630 (PMC5171641; doi:10.1038/srep39630)
Supplement: Supplementary Figures [file srep39630-s1.pdf]

Supplementary Figures

PRKAR1A is a functional tumor suppressor inhibiting ERK/Snail/E-cadherin pathway in lung adenocarcinoma

Shaoqiang Wang[1], Yuanda Cheng[1], Yingying Zheng[3], Zhiwei He[1], Wei Chen[1], Wolong Zhou[1], Chaojun Duan[2]*& Chunfang Zhang[1]*

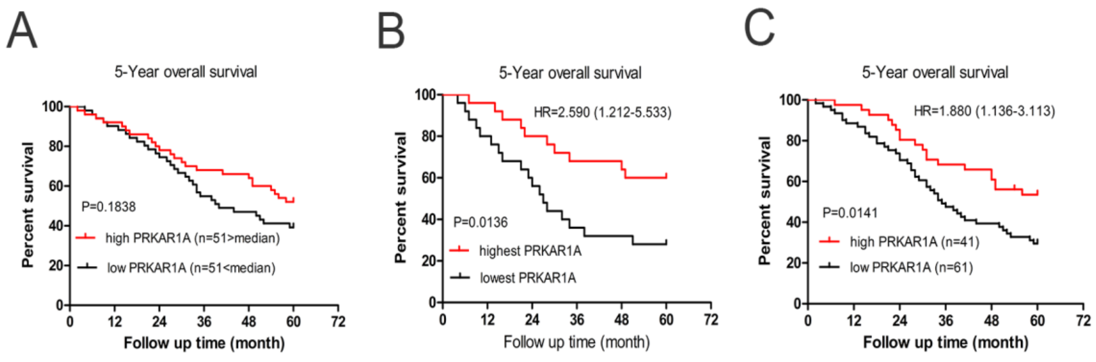

Fig S1. Kaplan-Meier analysis of 5-year overall survival. Kaplan-Meier analysis with (A) median (51 samples for each) and (B) highest (n=25) and lowest (n=25) quartiles of PRKAR1A mRNA expression for lung adenocarcinoma patients. (C) Kaplan-Meier analysis for lung adenocarcinoma patients with high (n=41) versus low (n=61) expression of PRKAR1A protein according to the results of Western blotting in Figure 1D.

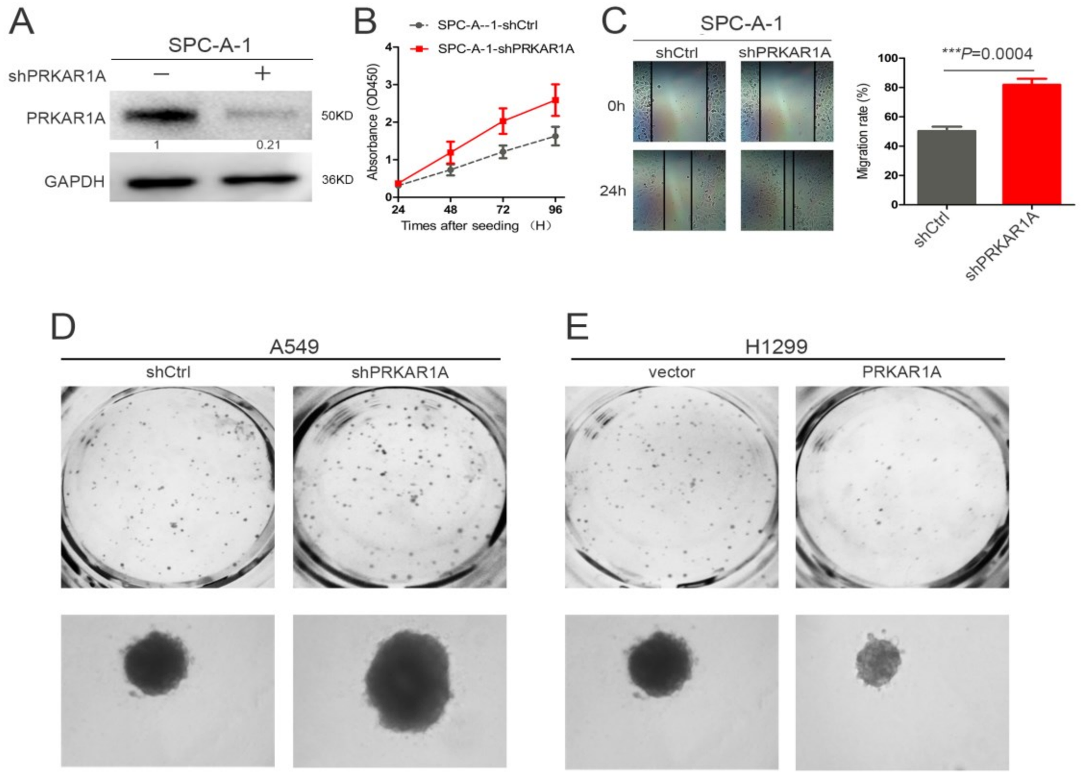

Fig S2. PRKAR1A inhibited proliferation and migration of lung adenocarcinoma cells. SPC-A-1 cells were stably transfected with shPRKAR1A. (A) Immunoblotting was conducted to determine the PRKAR1A protein expression in cells. (B) Cell growth was measured using CCK8 at various time points. (C) Cell migration was using scratch-wound assay Data were representative of three independent experiments. (D-E) Soft agar colony formation assay of PRKAR1A-knockdown (D) cells and PRKAR1A-overexpression (E) cells in 24-well dish (5×10^2 cells per well) for 2 weeks. Representative images in numbers (up) and mean size of agar colonies (down) were shown.

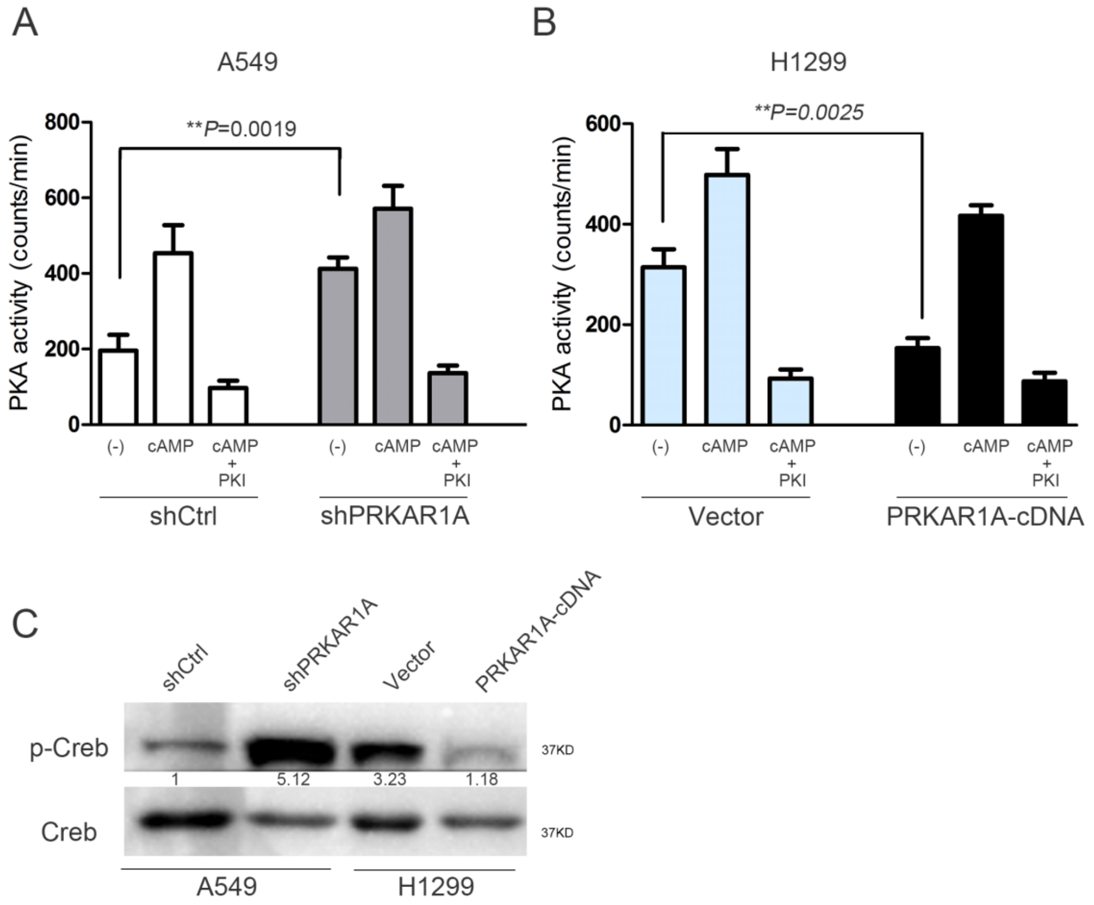

Fig S3. Changed PKA activity in vitro by PRKAR1A gene transfer. (A) and (B) Quantification of baseline (unstimulated) and total (cAMP-treated) PKA activity in A549 and H1299 cells. PKI co-treatment demonstrates PKA activity specificity. (A) Deletion of PRKAR1A results in elevated baseline PKA activity control to A549-shCtrl cells. (B) Restoration of PRKAR1A results in decreased baseline PKA activity control to H1299-vector cells. (C) The representative figure was illustrated of phosphorylated Creb (ser133) and total Creb protein levels in cells protein lysates

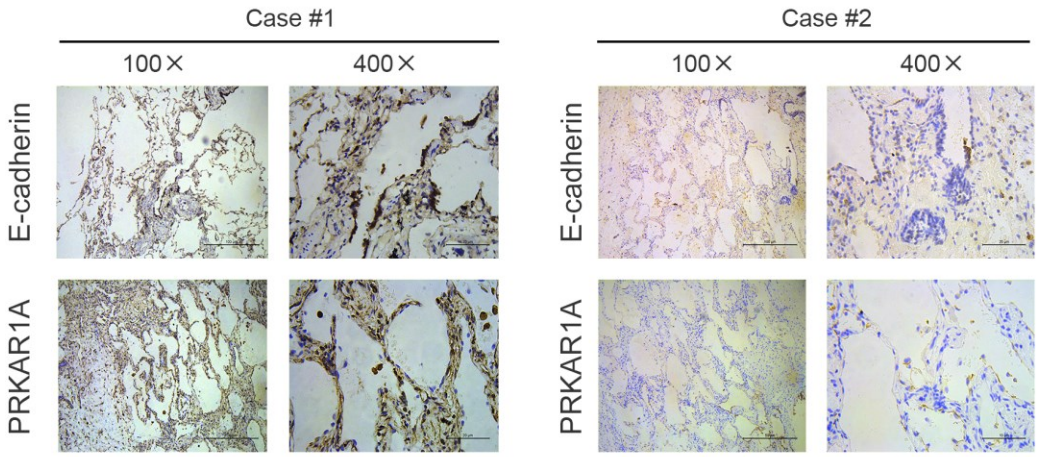

Fig S4. Representative images of immunohistochemical staining for PRKAR1A and E-cadherin in human normal lung tissues.

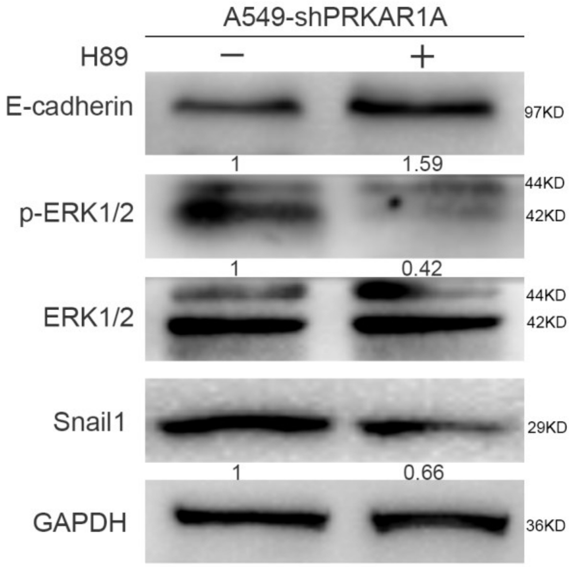

Fig S5. Relative E-cadherin, Snail1 and p-ERK1/2 protein expression analyzed with western blotting in H89 treated or not treated A549-shPRKAR1A cells. PKA inhibitor (H89) block phosphorylation of ERK1/2 and snail expression and accelerated E-cadherin expression in A549-shPRKAR1A cells. GAPDH was the loading control. The bands of p-ERK1/2 were normalized to total ERK protein.

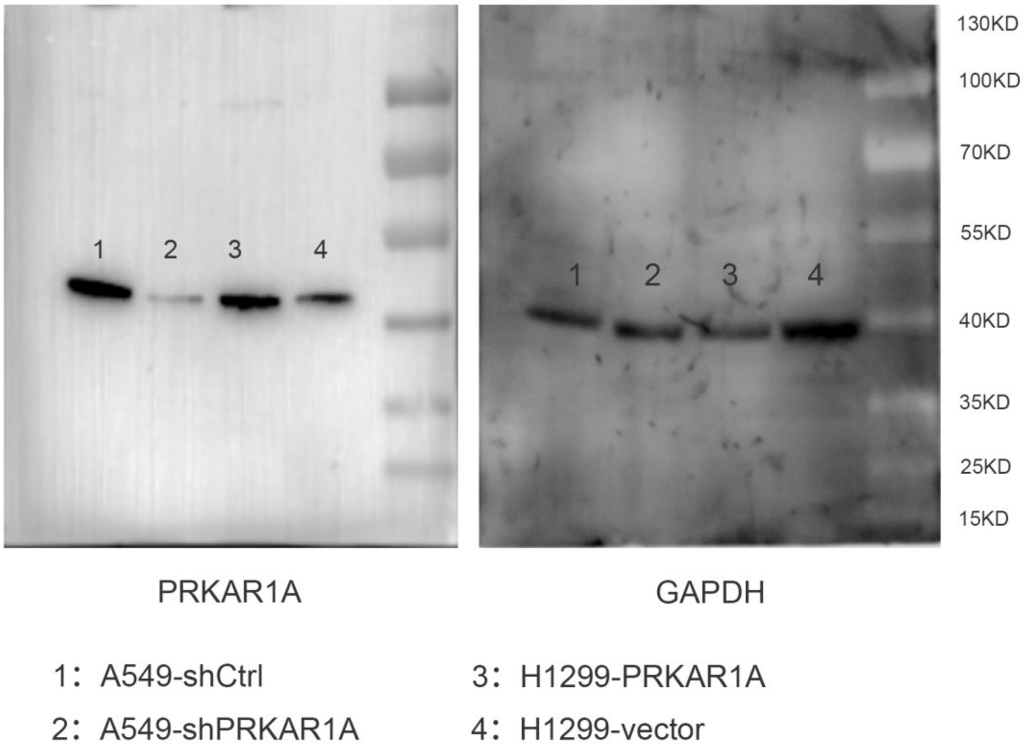

Fig S6. Full size of PRKAR1A band. The molecular weight of PRKAR1A (left) located between 40 KD and 55KD according to the marker protein. The molecular size of GAPDH (right) located between 35KD and 40KD.

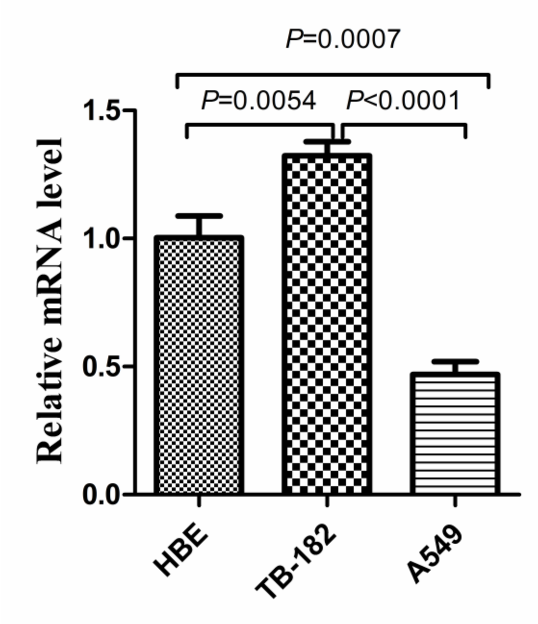

Fig S7. The relative PRKAR1A mRNA level. qRT-PCR analysis (performed in triplicate) of PRKAR1A in normal lung cell (HBE), lung squamous cell carcinoma cell (HTB-182), and human lung adenocarcinoma cell (A549).
